# Phase angle is a predictor of overall 5-year survival after head and neck cancer surgery

**DOI:** 10.1016/j.bjorl.2024.101482

**Published:** 2024-08-05

**Authors:** Neyara dos Santos Oliveira, Marcelo Leandro Santana Cruz, Ramon Silva de Oliveira, Tércio Guimarães Reis, Márcio Campos Oliveira, José de Bessa Júnior

**Affiliations:** a Universidade Estadual de Feira de Santana (UEFS), Feira de Santana, BA, Brazil; b Hospital Otorrinos/Multclin, Feira de Santana, BA, Brazil; c Santa Casa de Misericordia de Feira de Santana, Feira de Santana, BA, Brazil; d Universidade Estadual de Feira de Santana (UEFS), Departamento de Saúde, Feira de Santana, BA, Brazil

**Keywords:** Phase angle, Bioelectrical impedance analysis, Head and neck neoplasms, Prognosis, Survival

## Abstract

**Objective:**

To evaluate the ability of the variables Muscle Mass percentage (%MM), Body Fat percentage (%BF), Phase Angle (PhA), and Standardized Phase Angle (SPhA) to predict overall survival rates in individuals with surgically treated HNC.

**Methods:**

A prospective cohort study of surgically treated HNC patients was carried out between 2017 and 2022 at a cancer referral center in Feira de Santana, Bahia. All subjects were seen by an experienced dietitian the week before surgery. During this visit, patients had their sociodemographic and clinical data collected, as well as anthropometric measurements (weight and height) and BIA variables. In this study, postoperative overall 5-year survival was defined as the time in months between the date of surgery and the date of death, regardless of the cause of death. Patients were followed up after surgery until death.

**Results:**

Seventy-eight older adult patients were consecutively included. Patients had a median age of 65.5-years, were mostly men (83.3%), and most had low education levels (62.3%) and low household income, ranging from zero (19.2%) to the minimum wage (60.2%). They were mostly alcohol drinkers (91%) and tobacco smokers (87.2%). Glottic cancer was diagnosed in 48.7% of patients, with 44.8% of cases with stage-IV disease. Forty (51.2%) deaths were recorded in the follow-up period, with a median survival time of 39 months. Variables %MM, %BF, and SPhA were not significantly different between groups (alive vs. dead patients). PhA was considered an independent predictor of overall survival, with an accuracy of 69% (95% CI 0.57‒0.80). When comparing survival curves, patients with a preoperative PhA < 6.8° were more than twice as likely to die during follow-up (HR = 2.38; *p* =  0.02; 95% CI 1.14‒4.97).

**Conclusion:**

Out of the assessed BIA variables, preoperative PhA was considered a good predictor of overall survival after HNC surgery.

**Level of evidence:**

3.

## Highlights

• Preoperative phase angle was a good predictor of 5-year survival after HNC surgery.
• Phase angle measured at the onset of therapy may aid in the surgical treatment of HNC.
• Participants with phase angle values ≤ 6.8° had significantly worse survival times.

## Introduction

Head and Neck Cancer (HNC) is usually accompanied by impaired nutritional status and malnutrition before, during, and after treatment.^1^, ^2^, ^3^ Factors such as tumor location, adverse effects of treatment, and patient lifestyle contribute to reduced food intake, which results in weight loss and disease progression.^4^, ^5^

Nutritional follow-up is therefore important in all treatment phases. HNC patients are shown to have 5-year survival rates ranging from 30% to 60% in multiple studies,^6^, ^7^, ^8^ which are affected by personal, clinical, and healthcare quality factors.^9^, ^10^ As a result, identifying nutritional variables capable of predicting survival in this population is needed in order to provide these patients with better healthcare.

Studies looking into nutrition and prognosis in such patients are scarce. Bioelectrical Impedance Analysis (BIA), a noninvasive, simple, low-cost technique, is used to assess body composition, including Muscle Mass percentage (%MM), Body Fat percentage (%BF), total body water, and Phase Angle (PhA),^11^ and may yield variables capable of predicting survival.

BIA is a well-established tool for objectively assessing body composition and hence nutritional status^12^ in patients with various diseases, including cancer. BIA variables have been found to predict adverse clinical outcomes such as poor response to treatment, greater incidence of postoperative complications, worse quality of life in advanced cancer,^13^ and increased mortality in many clinical settings.^14^

There are few studies evaluating the role of BIA variables in predicting Overall Survival (OS) in surgically treated HNC cases. In recent years, PhA has been pointed out as a negative predictor of survival in HNC patients submitted mainly to chemotherapy and radiation therapy.^15^, ^16^, ^17^ In two studies, PhA was assessed in HNC patients treated with all three treatment options, surgery, radiation therapy, and chemotherapy.^18^, ^19^ Only one previously published study had a similar objective to the present work, namely the appraisal of preoperative PhA as a predictor of survival in HNC patients eligible for surgery.^20^

BIA is based on the principle that body tissues offer different degrees of opposition to the flow of predetermined, low-frequency electric current. Lean tissues are good conductors of electricity as they contain more water and electrolytes, which offer little resistance to the flow of electric current. On the other hand, fat, bone, and skin have low electrical conductivity, thus offering high resistance to the flow of electric current. Such differences in the electrical properties of biological tissues allow for the determination of body composition by BIA.^21^

BIA phase angle has been used as an indicator of homeostasis. It is a tool for nutritional diagnosis that has been increasingly employed in clinical practice. Lower PhA-values suggest reduced cell mass or impaired cell integrity, whereas higher PhA-values indicate a large number of undamaged cells. Recent research has validated PhA as a prognostic marker in critical patients, functioning as a measure of disease severity, a tool for functional assessment, and a general health indicator.^18^, ^21^

We then hypothesized that HNC patients diagnosed with malnutrition have worse survival rates. The aim of this project was to assess the ability of preoperative bioelectrical impedance variables (%MM, %BF, PhA, and SPhA) to predict overall 5-year survival after HNC surgery.

## Methods

A prospective cohort study was conducted between November 2016 and November 2022. Patients diagnosed with HNC who were treated at a cancer referral center located, were consecutively included.

Men and women aged 18 years or older who were submitted to en bloc surgery as the initial treatment of T2 to T4^22^ cancer of the oral cavity, larynx, oropharynx, hypopharynx, or nasopharynx were included in this study.

Patients diagnosed with dyslipidemia and taking lipid-lowering drugs or glucocorticoids, those with diseases affecting the normal metabolism of hepatic proteins, such as nephrotic syndrome, congestive heart failure, and cirrhosis, and individuals who did not provide written informed consent were excluded from the study. This project was approved by our institutional review board under the protocol nº 1.399.962.

All subjects were seen by an experienced dietitian the week before surgery. During this visit, patients provided written informed consent and had their sociodemographic and clinical data collected, as well as anthropometric measurements (weight and height) and BIA variables.

Body weight was measured with a Welmy® mechanical scale, with a 150 kg capacity and an accuracy of 100 g, while height was measured with an attached stadiometer with a maximum measuring capacity of 2.05 meters, conforming to the rules described by Lohman (1988).^23^

A *Biodynamics* BIA machine (model 450, version V.5.1) was used for bioelectrical impedance measurements, with a current of 800 μA at 50 kHz. Patient preparation before BIA measurements conformed to standardized rules proposed by Kyle and colleagues (2004).^24^ Body composition measurements including muscle mass (%), body fat (%), and phase angle were obtained.

The following equation was used to calculate SPhA: SPhA = [(measured PhA – mean PhA based on sex and age)/standard deviation of the reference population PhA]. Mean PhA and standard deviations proposed by Barbosa-Silva et al. (2008)^25^ were utilized as sex- and age-matched reference values for healthy adults. The 5^th^ percentile (point −1.65) was used to categorize SPhA values and establish the lower limit of normal for the healthy or risk-free population.

Nutrition support therapy consisting of an industrialized formula was initiated enterally in up to 12 hours after surgery as per protocol and patient specificities. During their hospital stay, patients were seen daily by a medical team in order to assess patient condition and monitor postoperative complications.

After discharge, patients were followed up on an outpatient basis by a multidisciplinary team, including dietitians, to keep track of their nutritional status, education on tube feeding, and changes in diet such as dietary supplementation and diet texture modifications.

In this study, postoperative overall 5-year survival was defined as the time in months between the date of surgery and the date of death, regardless of the cause of death. Death during follow-up was notified by the medical team itself or by the deceased patient’s family. Brazil’s National Civil Registry Information System was searched for patients’ names if they were lost to follow-up.^26^

Microsoft Excel was used for tabulation of data and GraphPad Prism, version 10.0.03 for Windows (San Diego, California, USA), was used for statistical analysis. Continuous and ordinal quantitative variables were presented as medians and interquartile ranges, while qualitative variables were expressed as absolute values and proportions.

The Student’s *t*-test and Mann-Whitney *U* test were used to compare continuous variables, while the Chi-Squared test and its variants were used to compare categorical variables. Receiver Operating Characteristic (ROC) curves were utilized to calculate and compare the overall accuracy of BIA variables, which was measured by the area under the ROC curve.

Kaplan-Meier curves were used to estimate overall survival probability over time and the log-rank test was used to compare survival distributions according to the stratification of variables. Ninety-five percent confidence intervals were employed as a measure of accuracy, and *p*-values < 0.05 (*p* < 0.05) were adapted to indicate statistical significance. Hazard Ratios (HR) were calculated and used to estimate effect size between subgroups.

Logistic regression analysis was performed to adjust for possible confounding variables (*p*-values < 0.05 in univariate analysis) and identify predictors of survival. Odds Ratio (OR) was calculated with a 95% Confidence Interval (95% CI).

## Results

A total of 78 subjects were included in this study, and most of them were male and had low education levels and low income. Forty (51.2%) deaths were recorded in the follow-up period, with a median survival time of 39 months. Detailed characteristics of the study population are shown in Table 1.

**Table 1:** Social and demographic characteristics of the study population.

Variable	Total (n = 78)
	n (%)
Age (years)*	65.5 (55–72)
Sex	
Male	65 (83.3%)
Female	13 (16.7%)
Education level	
Illiterate	17 (21.8%)
Primary school	49 (62.8%)
Secondary school	6 (7.7%)
Higher education	6 (7.7%)
Household income	
No income	15 (19.2%)
Up to the minimum wage	47 (60.2%)
One to 2 times the minimum wage	13 (16.8%)
≥ Two times the minimum wage	3 (3.8%)
Alcohol drinking	
No	7 (9.0%)
Yes	71 (91.0%)
Tobacco smoking	
No	10 (12.8%)
Yes	68 (87.2%)

About 50% of patients had laryngeal cancer. When comparing alive and dead patients, there was no statistically significant difference in primary tumor site. Among those who died 80% of the cases were locally advanced (*p* = 0.05). Detailed clinical characteristics of patients are summarized in Table 2.

**Table 2:** Preoperative clinical characteristics of the study population.

Variable	Total (n = 78)	Alive (n = 38)	Dead (n = 40)	*p*-value
	n (%)	n (%)	n (%)	
Primary tumor site				
Oral cavity	35 (44.8%)	16 (42.1%)	19 (47.5%)	0.19
Oropharynx	3 (3.8%)	3 (7.9%)	0 (0.0%)	
Larynx	40 (51.4%)	19 (50.0%)	21 (52.5%)	
Primary tumor subsite				
Tongue	10 (12.8%)	4 (10.5%)	6 (15.0%)	0.20
Buccal mucosa	2 (2.6%)	0 (0.0%)	2 (5.0%)	
Floor of the mouth	19 (24.3%)	9 (23.7%)	10 (25.0%)	
Lower gingiva	1 (1.3%)	1 (2.6%)	0 (0.0%)	
Base of the tongue	6 (7.7%)	5 (13.1%)	1 (2.5%)	
Aryepiglottic fold	2 (2.6%)	2 (5.2%)	0 (0.0%)	
Glottis	38 (48.7%)	17 (44.8%)	21 (52.5%)	
Clinical stage				
I	2 (2.6%)	2 (5.2%)	0 (0.0%)	0.05
II	24 (30.8%)	16 (42.1%)	8 (20.0%)	
III	17 (21.8%)	6 (15.8%)	11(27.5%)	
IV	35 (44.8%)	14 (36.8%)	21 (52.5%)	

When comparing alive and dead patients, there was no statistically significant difference in %MM and %BF, whereas PhA was significantly lower among deceased patients (*p* < 0.007). These findings are detailed in Table 3.

**Table 3:** Univariate analysis of BIA variables in alive and dead patients of the study population.

Variable	Total (n = 78)	Alive (n = 38)	Dead (n = 40)	*p*-value
Phase angle (PhA)	6.30 (5.38–7.03)	6.9 (5.6–7.7)	6.0 (5.2–6.6)	0.007
Muscle mass (%MM)	67.9 (56.6–76.1)	66.3 (57.3–74.9)	69.8 (51.7–77.0)	0.54
Body fat (%BF)	23.4 (16.9–31.5)	24.3 (18.4–32.0)	22.9 (14.9–30.4)	0.30

Regarding prognostic accuracy, PhA was found to have a discriminatory power of 69% (95% CI 0.57–0.80) to predict overall survival in the study sample, with the cutoff point of 6.8° yielding a sensitivity of 83% and a specificity of 53%, thus proving to be the best threshold.

**Figure 1:**
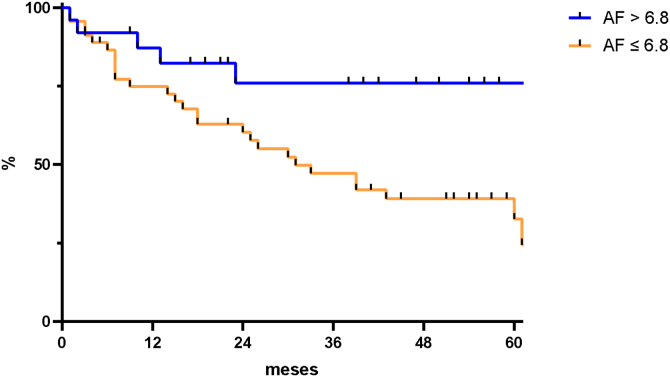
Kaplan-Meier curve comparing overall survival according to BIA Phase Angle (PhA) values in the study population.

Participants with PhA-values ≤ 6.8° were found to have significantly worse survival times when compared to those with PhA-values > 6.8° (HR = 2.38; *p* =  0.02; 95% CI 1.14‒4.97) (Fig. 1).

In a logistic regression model after adjustment for clinical staging, PhA remained associated with survival (*p* = 0.007; OR = 0.68; 95% CI 0.48–0.94).

In contrast, Standardized Phase Angle (SPhA) values were not found to be significantly different between groups with regard to survival in the follow-up period (HR = 0.88; *p* =  0.64; 95% CI 0.53‒1.46).

## Discussion

The epidemiology of HNC is well characterized in the literature. Male sex, age over 60 years, low income, low literacy, and a lifestyle marked by continued alcohol drinking and tobacco smoking are prominent sociodemographic features of this study cohort. These findings are consistent with those reported in other HNC studies.^3^, ^27^, ^28^, ^29^, ^30^

Population aging and the prolonged exposure to etiological agents such as alcohol and tobacco have been seen as one of the greatest challenges facing contemporary public health. The male population is more exposed to these risk factors, and low education levels are linked to poor socioeconomic conditions. When combined, these factors reinforce social disparities and affect the access to healthcare services, thereby impacting directly on the experience of cancer treatment.^30^

Cancer statistics such as incidence, mortality, and case fatality rates are extremely variable between Brazilian states. Assessing HNC survival has thus proved complex and challenging. HNC studies have shown 5-year survival rates to vary between 35% to 62.7%, a range that encompasses the findings of the present study (51.2%). This wide variation in survival rates may be explained by the strong association of worse treatment outcomes with lower human development index scores, greater social inequality, and lower local healthcare capacity in different states, as demonstrated in a Cancer Observatory report.^31^

At first sight, survival refers to health and life after cancer treatment until death. To further develop this concept, survival is proposed to include matters regarding a set of aspects centered on the individual-collective dimension and on the circumstances underlying healthcare access in order to tackle health problems to which patients are exposed, such as their support network comprising caregivers, friends, and family members.^32^ This certainly explains why individuals treated at different healthcare centers, with different experiences and different social and family interactions, end up having distinct survival times and quality of life.

Studies looking into survival of HNC patients with advanced disease have found median follow-up times to range from 32 to 42 months, a period similar to our finding (39-months). Importantly, survival time is significantly associated with patient nutritional status, and so it falls to a median of 13.4 to 19.6 months for those with impaired nutritional status.^16^, ^19^, ^20^

Phase angle was the only BIA variable found to be associated with survival in the present study. Few studies have looked into the role of BIA variables in predicting overall survival in surgical patients. In recent years, PhA has been classified as a negative predictor of survival in patients with HNC and squamous cell carcinoma mainly treated with chemotherapy and radiation therapy,^15^, ^16^, ^17^, ^33^, ^34^ while similar studies involving surgery as the initial treatment are still scarce.

PhA is correlated with cell quality, size, and integrity, thus indicating changes in body composition, cell membrane function, or health status. Lower PhA values may be associated with the onset or worsening of disease, cell death, or altered selective permeability of cell membranes. Higher PhA values, on the other hand, may be associated with a large number of intact cell membranes, i.e., greater body cell mass and good health status.^35^

The electrical properties of body tissues in HNC patients undergo changes, and so reductions in body fat, fat-free mass, and PhA are observed when compared to healthy individuals.^11^, ^36^ In cancer patients, low PhA-values might suggest cell membrane deterioration, which may lead to reduced overall survival. A positive association of PhA with nutritional status, BMI, fat-free mass, and serum albumin and transferrin levels has been shown in studies involving cancer patients with advanced disease (metastatic disease).^13^

These findings suggest changes in body composition observed in patients with HNC underlie such outcomes. Nutritional impairment in HNC is frequent and results from inadequate eating habits linked with alcohol and tobacco abuse, the tumor location in regions critical to the processes of chewing and swallowing, and the direct adverse effects of cancer treatment (radiation therapy, chemotherapy, and surgery) on food intake (odynophagia, dysphagia, trismus, use of nasoenteric tubes, among others).

PhA was found to be significantly lower (4.6° vs. 5.5°) in HNC patients when compared to healthy subjects in a review study,^37^ with a lower median PhA value than the one presented in this study (6.3°), which may be explained by the fact that we carried out BIA measurements before initiating antineoplastic therapy, which is known to contribute to nutritional status impairment, and hence to changes in body composition and cell membrane integrity.

In a study by Buntzel and colleagues (2019)^19^ with 42 HNC patients undergoing treatment (surgery with combined chemotherapy and radiation therapy), those with a PhA ≥ 5° were considered normally nourished and found to have a significantly longer survival time (*p* = 0.16) than malnourished patients (PhA < 5°), thus suggesting PhA is an indicator capable of predicting the impact of malnutrition on survival in this population. Survival was also found to be significantly shorter among patients with advanced HNC eligible for surgery and a PhA < 4.7° in a study conducted by Wladysiuk et al. (2016)^20^ with 75 participants (*p* = 0.04; HR = 1.88; 95% CI 1.00‒3.54).

PhA was found to have an accuracy of 69% to predict survival in HNC over a 5-year follow-up period in the present study. In another similar study, PhA was found to have an accuracy of 75% to predict survival over the same time span, considering three treatment options (surgery, chemotherapy, and radiation therapy).^18^

There has been an increase in the number of publications on the prognostic role of PhA in many fields over the last 2 years, but surgical patients with HNC remain understudied in this regard. The present study thus adds to the literature, but has limitations nonetheless, such as its single-center design and its limited sample size. Further research with similar methods is therefore needed before our results can be extrapolated to warrant the use of PhA as a predictor of survival in clinical practice.

## Conclusion

Out of the BIA variables assessed in this study, preoperative PhA was found to be a good predictor of survival over a 5-year period after HNC surgery, with an accuracy of 69%.

Prognostic significance of phase angle measured at the onset of therapy may aid in the treatment planning of patients with HNC, fostering quality of life and mitigating complications arising from impaired nutritional status.

## Funding

This research did not receive any specific grant from funding agencies in the public, commercial, or not-for-profit sectors. We also report no conflicts of interest regarding the publication of this article.

## Conflicts of interest

The authors declare no conflicts of interest.
